# Impairments in sensory-motor gating and information processing in a mouse model of *Ehmt1* haploinsufficiency

**DOI:** 10.1177/2398212820928647

**Published:** 2020-06-18

**Authors:** Brittany A Davis, François David, Ciara O’Regan, Manal A Adam, Adrian J Harwood, Vincenzo Crunelli, Anthony R Isles

**Affiliations:** 1 Neuroscience and Mental Health Research Institute and School of Biosciences, Cardiff University, Cardiff, UK; 2 MRC Centre for Neuropsychiatric Genetics and Genomics, School of Medicine, Cardiff University, Cardiff, UK

**Keywords:** Neurodevelopmental disorders, mouse model, startle and prepulse inhibition, auditory event–related potentials, NMDA-R hypofunction

## Abstract

Regulators of chromatin dynamics and transcription are increasingly implicated in the aetiology of neurodevelopmental disorders. Haploinsufficiency of *EHMT1*, encoding a histone methyltransferase, is associated with several neurodevelopmental disorders, including Kleefstra syndrome, developmental delay and autism spectrum disorder. Using a mouse model of *Ehmt1* haploinsufficiency (*Ehmt1*^D6Cre/+^), we examined a number of brain and behavioural endophenotypes of relevance to neurodevelopmental disorders. Specifically, we show that *Ehmt1*^D6Cre/+^ mice have deficits in information processing, evidenced by abnormal sensory-motor gating, a complete absence of object recognition memory, and a reduced magnitude of auditory evoked potentials in both paired-pulse inhibition and mismatch negativity. The electrophysiological experiments show that differences in magnitude response to auditory stimulus were associated with marked reductions in total and evoked beta- and gamma-band oscillatory activity, as well as significant reductions in phase synchronisation. The pattern of electrophysiological deficits in *Ehmt1*^D6Cre/+^ matches those seen in control mice following administration of the selective NMDA-R antagonist, ketamine. This, coupled with reduction of *Grin1* mRNA expression in *Ehmt1*^D6Cre/+^ hippocampus, suggests that *Ehmt1* haploinsufficiency may lead to disruption in NMDA-R. Taken together, these data indicate that reduced *Ehmt1* dosage during forebrain development leads to abnormal circuitry formation, which in turn results in profound information processing deficits. Such information processing deficits are likely paramount to our understanding of the cognitive and neurological dysfunctions shared across the neurodevelopmental disorders associated with *EHMT1* haploinsufficiency.

## Introduction

Post-translational modifiers of histone proteins influence chromatin dynamics and transcriptional regulation throughout development and are essential for the highly choreographed processes of lineage commitment and differentiation during neurodevelopment (Hirabayashi and Gotoh, 2010; Tyssowski et al., 2014). Perhaps unsurprisingly, exome sequencing studies and pathway analyses of genome wide association studies implicate genes encoding chromatin and transcriptional regulators in the aetiology of autism spectrum disorders (De Rubeis et al., 2014; Yuen et al., 2017), schizophrenia (Network and Pathway Analysis Subgroup of the Psychiatric Genomics Consortium, 2015) and severe developmental disorders (Deciphering Developmental Disorders Study, 2017; Singh et al., 2016). One such gene, implicated in several neurodevelopmental and neuropsychiatric disorders (Talkowski et al., 2012), is *EHMT1*, which encodes the histone H3 lysine 9 mono- and di-methyltransferase G9a-like protein (GLP).

Haploinsufficiency of *EHMT1* is the primary cause of the 9q34 subtelomeric-deletion syndrome, also known as Kleefstra syndrome (Kleefstra et al., 2005, 2006), a condition associated with intellectual disabilities, epilepsy, childhood hypotonia, facial dysmorphism, microcephaly, delays in reaching developmental milestones and behavioural problems such as aggressive outbursts and hypoactivity. Furthermore, analysis of copy number variants (CNVs) (Cooper et al., 2011) and a large exome sequencing study (Deciphering Developmental Disorders Study, 2017) have linked de novo mutations affecting *EHMT1* to severe developmental delay more generally. Finally, CNVs spanning *EHMT1* have also been associated with autism spectrum disorder (O’Roak et al., 2012) and schizophrenia (Kirov et al., 2012).

The importance of *Ehmt1* in brain function is supported by data from animal models that demonstrate a range of behaviour changes reminiscent of neurodevelopmental disorders including exploration and/or anxiety phenotypes (Balemans et al., 2010; Schaefer et al., 2009), and abnormal learning and memory (Balemans et al., 2013; Benevento et al., 2017; Iacono et al., 2018; Kramer et al., 2011). More recently, studies using rodent neuronal cultures and *ex vivo* slices (Benevento et al., 2016), and human induced pluripotent stem cell (iPSCs) (Frega et al., 2019), have shown that appropriate expression of *EHMT1* is required for the correct establishment and function neuronal networks. In human iPSCs, this neuronal network dysfunction is driven by the abnormal expression of *GRIN1* expression and enhanced NMDA-R signalling (Frega et al., 2019).

Here, we explore endophenotypes of relevance to psychiatric problems seen in those carrying mutation of *EHMT1*, using a mouse model of *Ehmt1* haploinsufficiency (*Ehmt1*^D6Cre/+^ mice). In order to reduce anatomical complexity and allow a more precise focus on the impact of *Ehmt1* haploinsufficiency during development, on later behavioural and neurophysiological parameters, we generated a forebrain-specific *Ehmt1* knockout mouse. Cre recombination was driven under the D6 promoter of the *Dach1* gene limiting *Ehmt1* heterozygous deletion to the forebrain, starting at embryonic stage 10.5 in the cortical vesicles (Van Den Bout et al., 2002; Machon et al., 2002). Specifically, we find deficits in sensory motor-gating and novel object recognition (NOR), and decreased anxiety. We then build upon recent *in vitro* and *ex vivo* evidence of abnormal neuronal networks and show a reduction in the magnitude of auditory evoked potentials in paired-pulse inhibition and mismatch negativity (MMN), providing in vivo electrophysiological evidence of an impairment in information processing in the *Ehmt1*^D6Cre/+^ mice. Gene expression data and pharmacological manipulation support the general idea that abnormal NMDA-R signalling in *Ehmt1*^D6Cre/+^ adult mouse contributing to the sensory-motor gating and information processing deficits.

## Materials and methods

### Animals

All procedures were conducted in accordance with the requirements of the UK Animals (Scientific Procedures) Act 1986, with additional ethical approval at Cardiff University.

In order to generate experimental cohorts, *Ehmt1*^fl/fl^ (Schaefer et al., 2009) male studs were paired in trios with one homozygous females carrying two copies of the Tg(Dach1-cre)1Krs/Kctt Cre transgene, maintained on a F1(C57BL/6J x CBA/Ca) background; and one wild-type F1(C57BL/6J x CBA/Ca) female. The *Ehmt1*^D6Cre/+^ mouse model was used in order to limit the effects of the deletion to the forebrain and hippocampus only and confounding effects of the non-CNS phenotypes, such as obesity (Balemans et al., 2010). Experimental cohorts were reared together and then weaned into mixed cages (2–5 per cage) of *Ehmt1*^D6cre/+^ (experimental line) and *Ehmt1*^fl/+^ mice (control line). All experimental subjects were male mice, and aged between 4 and 6 months during behavioural testing. A subset of the behavioural cohort was subsequently used in the electrophysiology experiments (7–8 months). Animals were housed 12-h-light/12-h-dark (lights on at 7 a.m.), and standard laboratory diet and water were available ad libitum throughout testing. Experimenters were blind to the genotype of animals during behavioural testing.

### Behaviour

All animals were initially subject to sensory-motor gating testing (*Ehmt1*^fl/+^, n = 25; *Ehmt1*^D6cre/+^ mice, n = 31). A subset of these was then subsequently tested on the rotor-rod (*Ehmt1*^fl/+^, n = 9; *Ehmt1*^D6cre/+^ mice, n = 18) and then elevated plus maze (EPM) and open field (OF). The remainder of the animals were subsequently tested on the NOR memory test (*Ehmt1*^fl/+^ mice, n = 16; *Ehmt1*^D6cre/+^ mice, n = 13).

#### Sensory-motor gating

Acoustic startle response (ASR) and prepulse inhibition (PPI) of the startle response were monitored using an SR-Lab apparatus (San Diego Instruments, San Diego, USA) modified for use in mice, according to published methods (McNamara et al., 2016). Briefly, animals were placed in a Perspex tube (internal diameter of 35 mm) and white noise stimuli were presented via a speaker. The amount of movement relayed as their startle response was measured as a piezoelectric measure converted to arbitrary startle units. The measurement used was the maximum startle (Vmax). Due to the effect of weight on this reflex movement measurement, all data were normalised for individual body weight. Pulse-alone trials consisted of a 40-ms 120-dB startle stimulus and a prepulse trial consisted of a 20 ms prepulse at 4, 8, or 16 dB above background and a 40-ms 120-dB startle stimulus, 70 ms after the prepulse. The stimuli were presented in a pseudorandom manner every 15 s. Whole body startle responses were recorded as the average startle during a 65-ms window timed from the onset of the startle pulse.

Percentage PPI score for each trial was calculated: (%PPI = 100 × (ASRstartle pulse alone − ASRprepulse + startle pulse)/ASRstartle pulse alone).

#### Rotarod testing

A rotarod task (Ugo Basile, Italy) was used to assess motor learning and co-ordination. This consisted of a rotating rod 30 mm in diameter, with five separated chambers 57 mm in width, with a rod elevation of 160 mm. Motor learning was assessed across six rotarod sessions; one morning session and one evening session, on three consecutive days. The rod speed accelerated incrementally from 5–50 r/min across the 300 s session. The main measure during training was latency to first fall. However, if the mice fell, they were continuously replaced on the rotating rod, until the full 300 s-session was over in order to prevent any confounds from arising from overall differences in time spent on the rotarod across sessions. In a separate test session on day four, the mice were given one 300 sec session at 10, 20, 30, 40, and 50 r/min consecutively in one morning session in order to assess motor coordination. Again, the latency to first fall was recorded for each animal at each speed.

#### NOR memory

The NOR test arena was a square 30 cm × 30 cm with 30 cm high, white Perspex walls. Four different, non-displaceable objects were used. All objects were white and selected for their equal appeal and available in triplicate to avoid the use of olfactory cues. In the habituation phase, 24 h prior to the task, each subject was allowed to explore the arena for 10 min in the absence of objects. In the acquisition phase, the subject was returned to the arena containing two identical sample objects (A, A’) and given 10 min to explore. After a retention phase, consisting of 15 or 90 min, the subject was returned to the arena with two objects, one identical to the sample and the other novel (A, B). During both the familiarisation and test phases, objects were located adjacent corners of the arena. The location of the novel object was counterbalanced. To prevent coercion to explore the objects, the subject was released in a third corner. Objects were cleaned with 70% ethanol wipes between sessions and object selection was randomised.

The main measure used was the Recognition Index (RI), indicating whether the animal investigated the novel object more than chance. This was calculated by the percentage of time spent investigating the novel object relative to the total object investigation (RI = T_N_/(T_N_ + T_F_) × 100). An RI significantly above chance or 50% indicates recognition of novelty and an RI equal to or below 50% indicates either no preference for the novelty or no recognition of novelty. Other parameters recorded were overall time spent with each object, and frequency of visits to the zones containing an object. Data were collected in 1-min time bins across the 10-min session by a camera linked to a computer with EthoVision Observer software (Noldus, Nottingham, UK).

### Electrophysiology

Adult male *Ehmt1*^D6Cre/+^ mice (n = 7) and *Ehmt1*^fl/+^ control cage-mates (n = 7) at 6–7 months of age were anesthetised with 2% isoflurane for stereotaxic electrode implantation. The electrode configuration used two bilateral frontal electrodes, one monopolar and one bipolar (2.7 mm anterior, 1.5 mm lateral, 1.2-deep relative to bregma); two bilateral hippocampal electrodes, one monopolar and one bipolar (2.7 mm posterior, 3 mm lateral, 2.2 mm deep relative to bregma); and one bipolar electrode in the auditory cortex (2.7 mm posterior, 4 mm lateral, 1.1-deep relative to bregma) as has been previously reported (Ehrlichman et al., 2008; Siegel et al., 2003) (for further details see Supplementary Figure 1). Due to animal loss, a subset of these was used in the MMN study; *Ehmt1*^D6Cre/+^ mice (n = 5) and *Ehmt1*^fl/+^ control cage-mates (n = 5).

Event-related potentials (ERPs) were obtained by averaging electroencephalography (EEG) traces centred at times 0 and 500 ms to 0 μV, respectively. For each trial, power was calculated using a complex Morlet wavelets w(t, f0) (Kronland-Martinet et al., 1987). The script used can be found at https://www.physics.lancs.ac.uk/research/nbmphysics/diats/tfr/. The wavelets have a Gaussian shape in the time domain (SD σt) and in the frequency domain (SD σt) around its central frequency ƒ0: w(t, f0) = A * exp(−t2/2 σt2) * exp(2iπf0t) with σf = 1/πσt. The wavelet family we used was defined by f0/σf = 1, with f0 ranging from 0.5 to 100 Hz in logarithmically distributed frequency steps (for full details, see Materials and Methods in Supplementary Information).

Auditory stimuli were generated using Spike2, version 7.2, and a Power1401 interface (CED, Cambridge, UK). Auditory stimulus was delivered with speakers positioned directly in front of each recording cage. Each mouse received an auditory, paired-pulse session and two sessions of MMN.

#### Paired pulse

Following Halene et al. (2009), each mouse received an auditory, paired-pulse session in which a single tone was presented at 1500 Hz and 90 dB (S1) followed by a 500-ms intra-trial interval and a second tone at 1500 Hz and 90 dB (S2). The tones were sinusoidal and 10 ms in duration. The inter-pulse interval between the two tones was 10 s. Each mouse received 1250 paired-pulse trials per recording session.

#### Mismatch negativity

The mice also received two sessions of the MMN protocol. Similar to Ehrlichman et al. (2008), the mice received 24 ‘standard’ tones at 90 dB and 1500 Hz and one ‘deviant’ tone at 90 dB and 2000 Hz. All tones were sinusoidal and 10 ms in duration and the intra-pulse interval between the 25 tones was 500 ms, while the inter-trial interval was 5 s. Each mouse was recorded for 360 trials in each of two sessions. In one session, 10 mg/kg of ketamine was administered, and in the second session, an equal volume of saline was administered. The dosage of ketamine was chosen based on previous work (Ehrlichman et al., 2008; Siegel et al., 2003). The within group design was counterbalanced by genotype and the order in which ketamine or saline sessions were administered. All recordings took place 5 min after intraperitoneal injections of either 10 mg/kg ketamine or the volume equivalent dose of saline. The waveform channels were filtered between 1 and 500 Hz.

### Gene expression

RNA was extracted from macrodissected hippocampi using the RNeasy micro kit (Qiagen) following the manufacturer instructions. A 96-well RT^2^ Custom Profiler PCR Array (CAPM12608, Qiagen, Manchester, UK) for mice was used. The custom genes list was generated based on GLP targets identified in the literature and tissue-specific relevance. For the qPCR array 1 μg of total RNA was used and manufacturer instructions were followed, for cDNA synthesis – RT^2^ First Strand Kit (Qiagen) and for the RT-PCR reaction – RT^2^ SYBR Green ROX qPCR Matermix (Qiagen). The average Ct values across the three housekeeping genes *B2m, B-actin* and *Gapdh* were used as endogenous controls. ΔCt values were generated by normalising to the geometric mean of the Ct values for these three housekeeping genes. All individual reactions were carried out in triplicate. Real-time qPCR data were visualised using the ΔΔCt method (Livak and Schmittgen, 2001).

### Statistics

All data were analysed using SPSS 20 (SPSS, Armonk, NJ, USA). The statistical differences between groups were analysed using independent samples t-tests, ANOVAs, or where appropriate Repeated Measures ANOVA (RP-ANOVAs). The main between-subject factor was GENOTYPE (*Ehmt1*^fl/+^ controls or *Ehmt1*^D6Cre/+^). The following within-subject factors were also analysed: TRIAL (Startle trial); PREPULSE (4, 8 or 16 dB); SESSION and SPEED (rotarod); PULSE (standard or deviant in the MMN). To check for normal distribution, Mauchly’s test of sphericity of the covariance matrix or Levene’s test for equality of variances were applied. The more conservative Huynh–Feldt corrections, with adjusted degrees of freedom are reported in cases in which test assumptions were violated. Non-parametric analyses were as follows. For the NOR task, the binomial distribution one-sample Kolmogorov–Smirnov (KS) test was applied to determine whether average RIs or SIs were significantly above chance (above 50%). For analysis of N1 amplitude, Wilcoxon rank-sum test was used. Statistical tests on the electrophysiological time-frequency data were performed using the permutation method with <1000 iterations (Westfall and Young, 1993) (see Materials and Methods in Supplementary Information for full details). For all comparisons, alpha value was set at p < 0.05.

## Results

### Impaired sensory-motor gating in *Ehmt1*^D6Cre/+^ mouse

**Figure 1.**
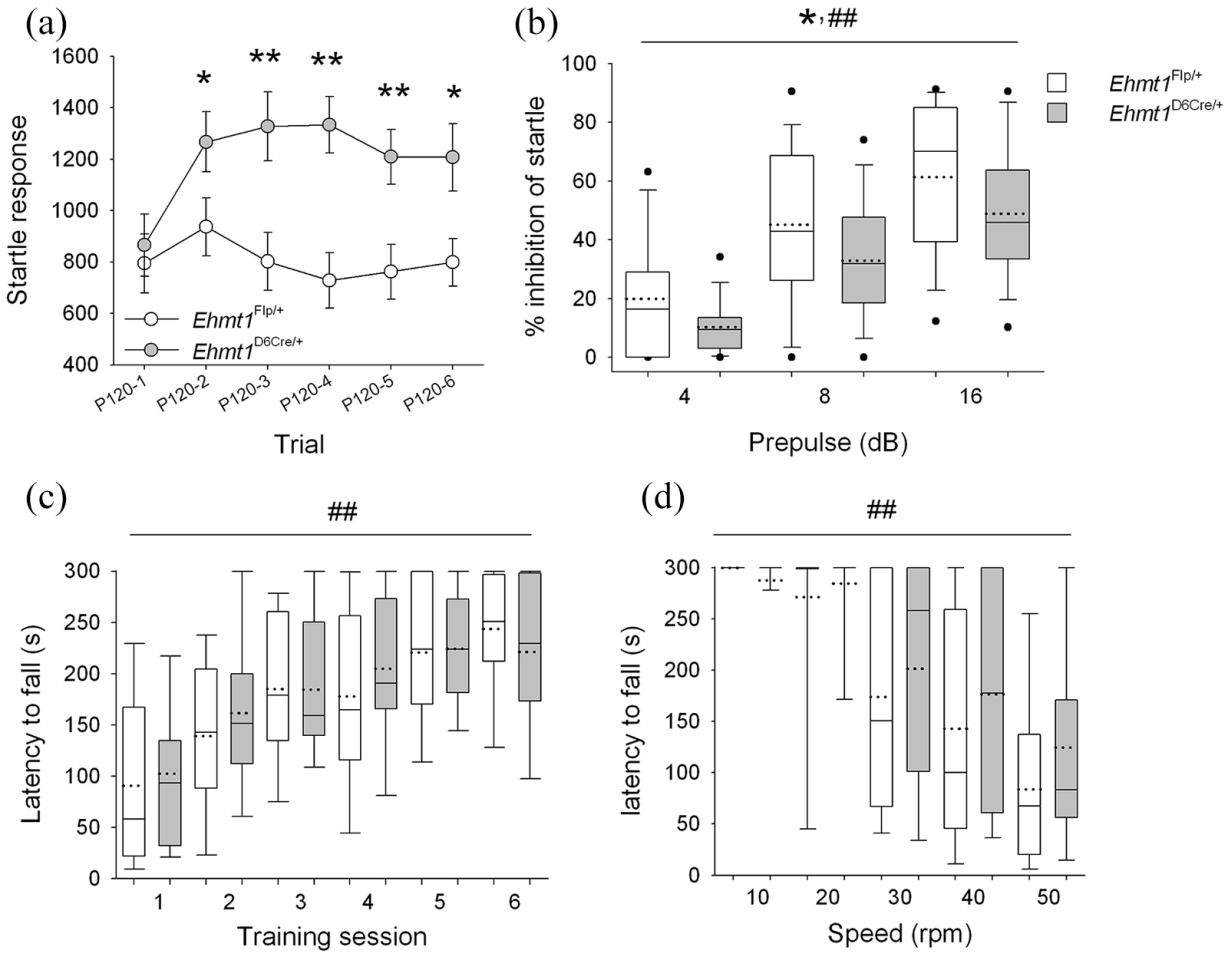
Sensory-motor gating, and motor learning and coordination. (a) The startle response after six consecutive 120-dB pulse stimulations show *Ehmt1*^D6Cre/+^ mice (n = 31) had a significantly higher overall maximum startle response in all trials. (b) Prepulse inhibition of the startle response generally increased with the increasing volume of the prepulse as expected. However, *Ehmt1*^D6Cre/+^ displayed a 20%–40% reduction in PPI of startle relative to *Ehmt1*^Fl/+^ mice (n = 25). (c) Both *Ehmt1*^Fl/+^ (n = 9) and *Ehmt1*^D6Cre/+^ mice (n = 18) improved motor ability on the rotarod as evidenced by an increase in latency to fall across 6 training sessions. There was no also difference in genotype during training. (d) In a probe test, where rotarod speed accelerated throughout the session, there was a general decrease in the latency to fall as the speed increased, but again no difference between genotypes. Data are mean ± SEM, or box plots showing median (solid line), mean (dotted line) and 10th, 25th, 75th and 90th percentiles. * represents main effect of GENOTYPE p < 0.05; ## represents main effect of within-subject factor (TRIAL, PREPULSE, SESSION or SPEED).

The ASR and PPI of this response, were used to examine sensory-motor function in the *Ehmt1*^D6Cre/+^ mice. Over the course of six consecutive auditory pulse (120 dB) startle trials, *Ehmt1*^D6Cre/+^ mice had, on average, twice the startle response compared to *Ehmt1*^fl/+^ mice (Figure 1(a); ANOVA, main effect of GENOTYPE, F_1,53_ = 6.86, p = 0.01). A significant interaction between GENOTYPE and TRIAL (F_3.87,201.0_ = 2.48, p = 0.03) indicated different patterns of startle reactivity and habituation relative to *Ehmt1*^fl/+^ mice. Post hoc analysis revealed that there was an equivalent startle response to the initial trial between genotypes (p *=* 0.68), but on average *Ehmt1*^D6Cre/+^ mice showed a significantly enhanced startle response in all consecutive trials (trial 2, p *=* 0.05; trial 3, p *=* 0.01; trial 4, p *=* 0.001; trial 5, p *=* 0.01; and trial 6, p *=* 0.02).

As expected, increasing the prepulse volume led to a linear increase in the inhibition of the startle in both groups (Figure 1(b); ANOVA, main effect of PREPULSE, F_1.49,80.35_ = 73.26, p *=* 0.001). However, there was a 20%–40% PPI reduction in the *Ehmt1*^D6Cre/+^ mice relative to *Ehmt1*^fl/+^ cage-mates (Figure 1(b); ANOVA main effect of GENOTYPE, F_1,54_ = 5.54, p = 0.022), suggesting that, in addition to an enhanced startle response, the mutant mice were also impaired in the normal PPI response

### Normal motor function in the *Ehmt1*^D6Cre/+^ mouse

Altered sensory-motor gating in *Ehmt1*^D6Cre/+^ mice was not due to any gross deficits in motoric competence. Training on the rotarod test indicated normal learning with repeated sessions, with latency to fall reducing across training sessions (Figure 1(c); ANOVA, main effect of SESSION, F_5,130_ = 21.33, p < 0.001), but there was no difference in GENOTYPE (F_1,26_ = 0.12, p = 0.73) and no interaction between training SESSION and GENOTYPE (F_5,130_ = 0.67, p = 0.65). Moreover, in an accelerating rotarod probe test of motor coordination, latency to fall decreased as the speed increased (Figure 1(d); ANOVA, main effect of SPEED, F_4,104_ = 38.38, p < 0.001), but there was no difference between GENOTYPE (F_1, 26_ = 0.67, p = 0.42) or interaction between SPEED and GENOTYPE (F_4,104_ = 0.65, p = 0.63).

### Reduced anxiety in the *Ehmt1*^D6Cre/+^ mouse

**Figure 2.**
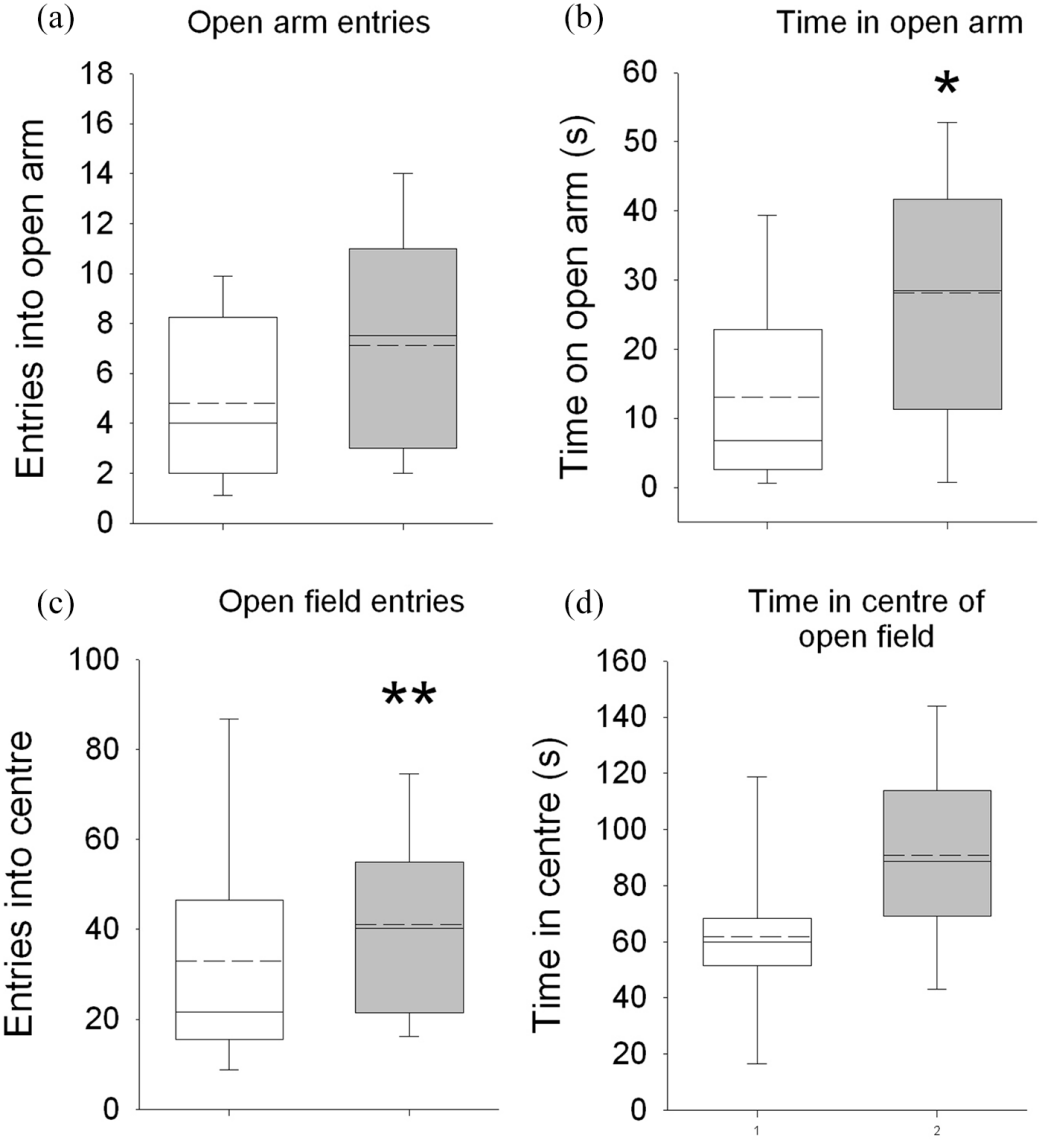
Behaviour in the elevated plus maze and open field. On the EPM, *Ehmt1*^D6Cre/+^ mice (n = 17) showed a pattern of behaviour consistent with reduced anxiety relative to *Ehmt1*^Fl/+^ mice (n = 9), including increased open arm entries (a) and increased time on time on the open arm (b), although only the latter was statistically different from controls. Convergent evidence of a reduced anxiety phenotype was seen in the OF test. Here, *Ehmt1*^D6Cre/+^ mice made significantly more entries into the centre of the OF (c) and spent more time in the inner zone (d), although this did not reach significance. Box plots showing median (solid line), mean (dashed line) and 10th, 25th, 75th and 90th percentiles. * represents main effect of GENOTYPE p < 0.05; ** p < 0.01.

A number of measures in the elevated plus maze and open field tests indicated that *Ehmt1*^D6Cre/+^ mice have a reduced anxiety phenotypes. On the EPM (Figure 2(a) and (b)), *Ehmt1*^D6Cre/+^ mice spent on average twice as long as *Ehmt1*^Fl/+^ cage-mates on the open-arm (t_26_ = −2.08, p = 0.04) and made on average 40% more entries into the open arm of EPM more than controls, although this did not reach statistical significance (t_26_ = −1.52, p *=* 0.14). On the OF test (Figure 2(c) and (d)), *Ehmt1*^D6Cre/+^ mice made on average 25% more entries in the inner zone of the OF (t_26_ = −3.21, p = 0.004) and spent 50% more time in the inner zone than *Ehmt1*^Fl/+^ mice, although this did not reach statistical significance (t_26_ = −1.86, p = 0.07).

### Impaired NOR in *Ehmt1*^D6Cre/+^ mouse

**Figure 3.**
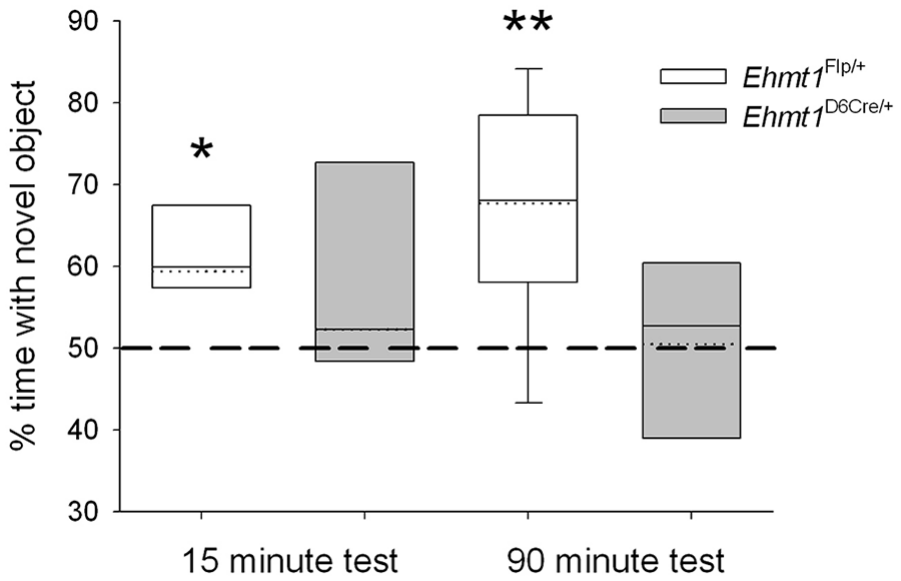
Novel object recognition. In both the 15-min and 90-min retention tests of the NOR task *Ehmt1*^fl/+^ control mice (n = 16) explored the novel object more than chance. However, exploration of the novel object by *Ehmt1*^D6Cre/+^ mice (n = 13) was not significantly above chance, for either the 15- or 90-min retention trials indicating a deficit in declarative memory. Data are box plots showing median (solid line), mean (dotted line) and 10th, 25th, and 75th and 90th percentiles (dots represent 5th and 95th percentile). * represents main effect of GENOTYPE p < .05; ** p < .01.

The NOR task takes advantage of the preference of rodents to attend to new objects in their environment as a means for testing declarative memory (Antunes and Biala, 2012). Here, two inter-trial intervals were used: half of the cohort was examined following a 15-min delay between the initial object exposure and the test trial, and half the cohort was examined after a 90-min delay. In the test phase, as expected the *Ehmt1*^fl/+^ control mice explored the novel object significantly more than chance (50%) in both the 15-min and 90-min retention trials (Figure 3; Kolmogorov–Smirnov test (KS), 15 min, 60%, p = 0. 02; 90 min retention trial, 67%, p = 0.002). However, exploration of the novel object by *Ehmt1*^D6Cre/+^ mice was not significantly above chance, at either 15-min (52%, p = 0.97) or 90-min retention trial (50%, p = 0.44).

This difference at test was not due to differences during the habituation phase, as indicated by overall exploration time of the object that was replaced by a novel object at test (*Ehmt1*^fl/+^ mean = 114 s, ± SEM = 15; *Ehmt1*^D6Cre/+^ mean = 113 s ± SEM = 12; ANOVA, no main effect of GENOTYPE, F_1,28_ = 0.031, p = 0.86).

### Electrophysiological measurements of paired-pulse auditory evoked potentials

**Figure 4.**
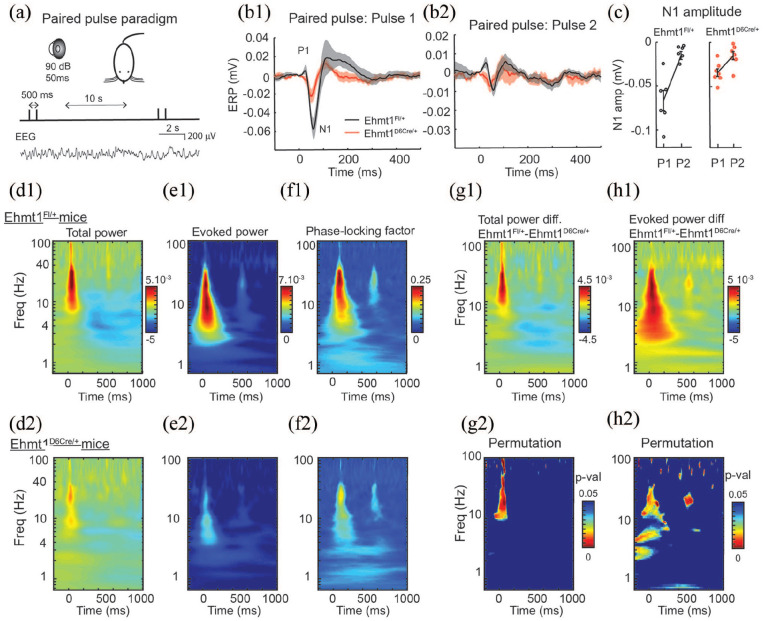
AEP paired-pulse measurements. (a) Schematic showing the paired-pulse stimulus paradigm. (b1) Grand average ERP waveform after pulse 1 (S1) in *Ehmt1*^D6Cre/+^ (red) *and Ehmt1*^fl/+^ (black). The P1 and N1 components are indicated on the waveform. (b2) The waveforms for pulse 2 (S2); (c) N1 amplitude for *Ehmt1*^D6Cre/+^ (n = 7) and *Ehmt1*^fl/+^ (n = 7) mice. (d1 and d2) Normalised total power (in time-frequency plot) of both S1 (time 0) and S2 (time 500 ms) for *Ehmt1*^fl/+^ (d1) and *Ehmt1*^D6Cre/+^ (d2). Note the early increase in the beta/gamma range (10–100 Hz) and the late decrease in the delta range (~4 Hz) and the reduction of both components in the *Ehmt1*^D6Cre/+^. (e1 and e2) Evoked power time-frequency plots for the same data set; (f1 and f2) phase locking factor for the same data set; (g1) difference of total power between *Ehmt1*^fl/+^ and *Ehmt1*^D6Cre/+^; (g2) Statistical significance heat map based on permutation tests of total power are indicated by the colour scale (p < 0.05); (h1) difference of evoked power between *Ehmt1*^fl/+^ and *Ehmt1*^D6Cre/+^ mice; and (h2) statistical significance between evoked power of *Ehmt1*^fl/+^ and *Ehmt1*^D6Cre/+^ mice (spurious spots above 50 Hz are due to occasional 50 Hz noise contamination in some of the recordings). Data shown as mean ± SEM.

In order to gain an insight into the neural changes underpinning the deficits in sensory-motor gating seen in *Ehmt1*^D6Cre/+^ mice, we used electrophysiological methods. A subset of the behavioural cohort was then subject to EEG recording to measure auditory evoked potentials (AEPs). AEPs are voltage fluctuations time-locked to an auditory event used for brain dysfunction clinical diagnosis (Luck et al., 2011). We used a paired-pulse paradigm in which two pulses were delivered back-to-back with a 500-ms interval between the first stimulus (S1) and the second stimulus (S2) (Figure 4(a)). The grand average waveforms show a stereotypic maximal positive deflection (P1) and maximal negative deflection (N1) (Figure 4(b1)). *Ehmt1*^D6Cre/+^ mice had a nearly twofold lower N1 amplitude response after the S1 (Figure 4(b1)) but not after S2 (Figure 4(b2)). In addition, *Ehmt1*^D6Cre/+^ mice had a significant reduction in the S2/S1 ratio for the N1 component (Wilcoxon, rank sum = 64, p = 0.018), what is considered an electrophysiological measurement of sensory gating (Figure 4(c)). During peak activation of the paired-pulse experiment we observed an increase in total power across high-frequency bands from 10–100 Hz in both groups of mice (Figure 4(d1) and (d2)). The difference time-frequency plot and the permutation test between *Ehmt1*^D6Cre/+^ and *Ehmt1*^fl/+^ control mice however, revealed that the distributed peak that occurred approximately 40 ms after the S1 pulse, a time corresponding with the N1, was significantly greater in the beta (13–30 Hz) and gamma (30–70 Hz) frequency bands in the *Ehmt1*^fl/+^ control mice (Figure 4(g1) and (g2)). The late (>200 ms after the S1 pulse) decrease in the delta frequency band (~4 Hz) prominent in *Ehmt1*^fl/+^ (Figure 4(d1)–(g1)) was not different from *Ehmt1*^D6Cre/+^ (Figure 4(g2)).

Measurements of evoked power, EEG power which is phase-locked with the event onset across trials, demonstrated increases in the delta (~4 Hz), beta (13–30 Hz) and low gamma (here ~40 Hz) band responses in both groups of mice approximately ~30–50 ms after the S1 (Figure 4(e1) and (e2)). Again, permutation tests revealed a reduction in evoked power in *Ehmt1*^D6Cre/+^ mice after both the S1 and ~40 ms after the S2 pulse (Figure 4(h1) and (h2)). In complement, we measured the phase locking factor (PLF), which provide a measurement of trial-to-trial reliability across the frequency domain (Goffin et al., 2014). To extract the PLF, magnitude information is transformed to 1 and averaged so that the phase angle with respect to event onset is extracted (Roach and Mathalon, 2008). Values between zero and one are obtained, in which one reflects perfect synchrony of phase angles across trials. *Ehmt1*^D6Cre/+^ mice did not show PLF values above .17 at any point, while control mice show nearly twofold PLF synchrony (+.25) at ~40 ms post-S1 pulse, between ~20 and 40 Hz. Overall *Ehmt1*^D6Cre/+^ mice demonstrated reduced PLF (Figure 4(f1) and (f2)).

### Electrophysiological measurements of MMN AEPs

**Figure 5.**
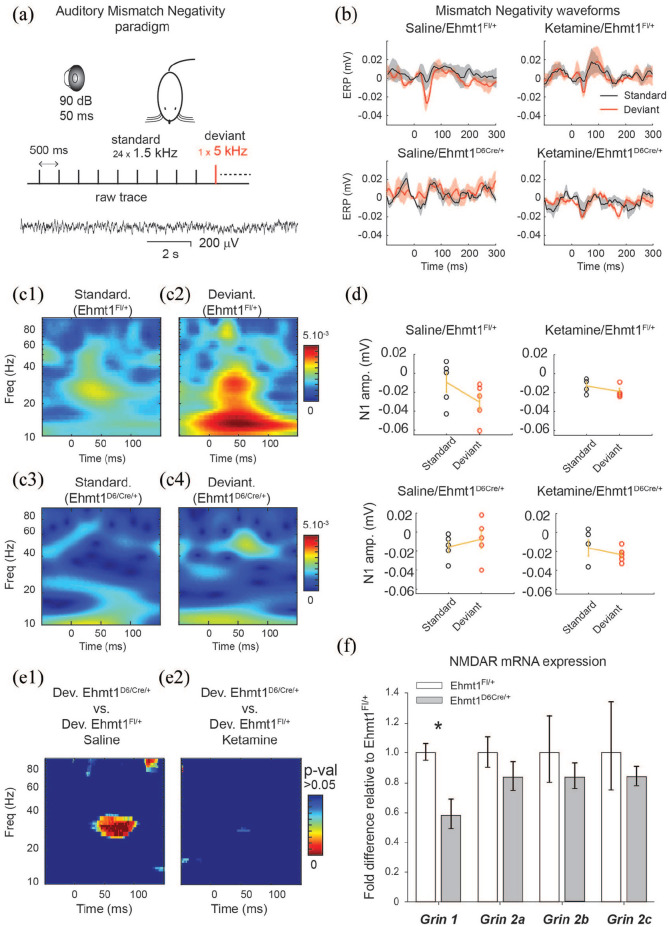
AEP mismatch negativity phenotypes and NMDA mRNA expression. (a) Mismatch negativity stimulus. (b) Grand average ERP waveforms (shaded areas) after the saline or ketamine conditions in the in *Ehmt1*^fl/+^ (n = 5) and *Ehmt1*^D6Cre/+^ (n = 5), for the deviant (red) and standard (black) tone for each condition. (c1 and c2) Time-frequency plot of evoked power for the standard and deviant pulse in *Ehmt1*^fl/+^ mice after saline injection. (c3 and c4) Time-frequency plot of evoked power for the standard and deviant pulse in *Ehmt1*^D6Cre/+^ mice following saline injection. (d) N1 amplitude peak amplitude corresponding to each condition. (e1) Permutation test showing heat map of p-values for the difference of distribution between deviant tone maps of saline treated *Ehmt1*^fl/+^ (c2) and *Ehmt1*^D6Cre/+^ (c4). (e2) Permutation test showing heat map of p-values for the difference of distribution between deviant tone maps of ketamine treated *Ehmt1*^fl/+^ (c2) and *Ehmt1*^D6Cre/+^ (c4). (f) NMDA-R subunit mRNA expression in hippocampal samples (*Ehmt1*^fl/+^ n = 4, *Ehmt1*^D6Cre/+^ n = 8). Data shown are mean ± SEM. * represents main effect of GENOTYPE p < 0.05.

The MMN response is elicited when a qualitative feature of the stimulus does not match the pattern in a previous series (Light and Braff, 2005) (Figure 5(a)). One core feature of MMN is the importance of NMDA receptor function. For instance, non-competitive NMDA-R antagonists, like ketamine and the selective antagonist of the NR2B NMDA subunit, CP-101,606 reduce MMN amplitude (Sivarao et al., 2014), the level of which predicts the magnitude of psychotic experiences in response to these drugs (Umbricht et al., 2002). Therefore, in order to probe this function further here, mice were given either saline or 10 mg/kg of ketamine prior to the MMN test.

In saline-treated animals, there was a difference between *Ehmt1*^D6Cre/+^ and *Ehmt1*^fl/+^ mice in response to the deviant pulse (Figure 5(c)–(d)) as indicated by analysis of the N1 amplitude that showed an interaction between GENOTYPE and PULSE (repeated measures ANOVA, F_1,8_ = 12.70, p = 0.007). In the standard pulse condition *Ehmt1*^fl/+^ mice, amplitude corresponded to an increase in ~10–40 Hz evoked potential approximately 30–50 ms post-stimulus (Figure 5(c1)–(c2)). In the deviant pulse condition, *Ehmt1*^fl/+^ mice not only show an increase in ~10–40 Hz evoked potential, but also one with greater peak latency, at 40–70 ms post-stimulus (Figure 5(c2)). In contrast, *Ehmt1*^D6Cre/+^ mice treated with saline showed no pattern of change in the amplitude response for either standard or deviant pulses.

However, following 10 mg/kg of ketamine administration, the difference in the N1 amplitude between the standard and deviant pulse was absent for both genotypes (Figure 5(b) and (d)), as indicated by a lack of interaction between GENOTYPE and PULSE (repeated measures ANOVA, F_1,7_ = 0.011, p = 0.921). Further analysis underlined this finding. Permutation tests after saline administration for the deviant-only condition demonstrates a difference between *Ehmt1*^fl/+^ controls and *Ehmt1*^D6Cre/+^ wavelet transforms, at 30 Hz between 50 and 80 ms post-stimulus (Figure 5(e1)), driven by the higher amplitude response in the *Ehmt1*^fl/+^ control mice. In contrast, after ketamine administration, the difference in deviant response between *Ehmt1*^fl/+^ controls and *Ehmt1*^D6Cre/+^ mice was abolished (Figure 5(e2)).

### Altered NMDA-R expression in *Ehmt1*^D6cre/+^ mutant mice

In light of similarity in basal *Ehmt1*^D6Cre/+^ electrophysiological phenotype and the electrophysiological phenotype in *Ehmt1*^fl/+^ control mice following ketamine administration, we assessed the NMDA system in *Ehmt1*^D6Cre/+^ mice. RT qPCR was used to examine the mRNA levels of NMDA-R subunits *Grin1, Grin2a, Grin2b* and *Grin2c* in the adult hippocampus. Expression of *Grin1*, the gene encoding NMDA NR1 subunit, was significantly reduced by 40% on average in *Ehmt1*^D6cre/+^ mice (Figure 5(f); t = −3.07, p = 0.014). No difference was seen in expression of other NMDA-R subunits examined (Figure 5(f) *Grin2a*, t = −1.02, p = 0.331; *Grin2b*, t = −0.814, p = 0.435; or *Grin2c*, t = −0.71, p = 0.498). Furthermore, there were no genotype differences in AMPA-R subunit gene expression examined (see Supplementary Figure 4).

## Discussion

Recent studies in human genomics strongly implicate a number of genes found to regulate chromatin dynamics in the aetiology of neurodevelopmental disorders from developmental delay (Singh et al., 2016), to autism spectrum disorders (De Rubeis et al., 2014; Yuen et al., 2017), and schizophrenia (Network and Pathway Analysis Subgroup of the Psychiatric Genomics Consortium, 2015). *EHMT1* is one such gene, associated with both neurodevelopmental and neuropsychiatric disorders. Here, we examined behavioural and electrophysiological correlates of information processing in a mouse model of *Ehmt1* haploinsufficiency, specifically in the forebrain. We found that *Ehmt1*^D6Cre/+^ mice have sensorimotor and auditory gating deficits, reduced anxiety, and learning and memory deficits, in the absence of generic deficits in motor function. *Ehmt1*^D6Cre/+^ mice also showed a number of abnormalities in electrophysiological measurements including a reduced magnitude of AEPs after paired-pulse inhibition and MMN tasks, reduced evoked and total power in high-frequency bands, and reduce PLF. Overall, these data indicate that *Ehmt1*^D6Cre/+^ mice show deficits in sensory motor gating and information process, possibly related to abnormal NMDA-R functioning.

*Ehmt1*^D6Cre/+^ mice displayed decreased anxiety in a number of measures across a two separate tests, namely, the EPM and OF. Although these findings in our model were not confounded by impaired motor competence as indicated by normal behaviour and learning on the rotarod test, not all measures on both the EPM and OF reached statistical significance. It may be that a more robust assessment of anxiety in this model would be achieved using a unified score from a number of separate measures (Harrison et al., 2020). Generally, however, these findings of decreased anxiety are consistent with previous studies of *Ehmt1* haploinsufficient models (Balemans et al., 2010; Iacono et al., 2018), although other models, specifically a CamKII-driven full *Ehmt1* deletion shows decreased anxiety (Schaefer et al., 2009). This indicates that the relationship between *Ehmt1* function and anxiety behaviour probably depends on extent, timing and location of the genetic lesion.

*EHMT1* is a risk gene associated with a number of neurodevelopmental and psychiatric disorders (Talkowski et al., 2012), thus it was important for this work to focus not only on translational phenotypes but those that overlap traditional diagnostic boundaries. Several prominent features shared by these disorders are associated with deficits in attention and gating, or filtering out intrusive stimuli (Belmonte et al., 2004; Javitt, 2009; Orekhova et al., 2008; Perry et al., 2007). Behaviourally, the *Ehmt1*^D6Cre/+^ mice showed evidence of this in an acoustic startle test. Consistent with previous findings in other *Ehmt1* haploinsufficiency models (Balemans et al., 2013; Iacono et al., 2018), sensory motor gating deficits were manifest by both a greatly enhanced ASR and a decreased PPI of startle.

Information processing deficits are another common phenotype associated with *EHMT1* risk populations. Accordingly, *Ehmt1*^D6Cre/+^ mice were examined on their performance in the NOR task, a paradigm where an animal’s ability to remember a previously encountered object is indicated by an increased willingness to explore the novel object over the familiar object. The *Ehmt1*^D6Cre/+^ mice showed no evidence of NOR, even with only a 15-min interval after habituation. Importantly, this was not due to reduced interest in exploration or exposure to the familiar object, as time spent in exploration was equivalent across *Ehmt1*^fl/+^ control and *Ehmt1*^D6Cre/+^ mutant mice. Similar to the acoustic startle and PPI phenotype, deficits in a NOR paradigm have been seen in other *Ehmt1*^+/−^ mice (Balemans et al., 2013; Iacono et al., 2018). This convergence across models and laboratories suggests that both sensory motor-gating and information processing deficits are key behavioural features of *Ehmt1* haploinsufficiency.

Given the robustness of behavioural deficits in sensory motor-gating and information processing seen in models of *Ehmt1* haploinsufficiency, we explored this further by examining electrophysiological measures of these domains. *Ehmt1*^D6Cre/+^ had reduced N1 amplitude in response to stimulus 1 (S1). Furthermore, *Ehmt1*^D6Cre/+^ mice had a significant reduction in the S2/S1 ratio for the N1 component, an electrophysiological correlate of sensory gating.

Evidence of information processing deficits in the *Ehmt1*^D6Cre/+^ mice was provided by the reduced response in a MMN paradigm. The decreased amplitude responses in these AEP measurements meanwhile corresponded to significant reductions in total and evoked power in high-frequency bands. Such reductions may be particularly insightful, as numerous studies report disruptions in gamma-band activity across neurodevelopment (Kwon et al., 1999; Orekhova et al., 2008), neuropsychiatric (Tatard-Leitman et al., 2015) and neurodegenerative diseases (Iaccarino et al., 2016). Whether reduced gamma activity is actually directly associated with disease pathologies across these patient populations remains largely unknown. Recently, however, reductions in gamma-band activity were found to precede the onset of plaque formations and cognitive decline in a mouse model of Alzheimer’s disease. Meanwhile, the stimulation of fast-spiking interneurons in the gamma frequency range (~40 Hz), a way to boost gamma-band activity (Cardin et al., 2009), led to the reduction in the plaque forming amyloid isoforms and attenuated plaque load in ageing mice (Iaccarino et al., 2016). In a more functional assessment, the induction of long-term potentiation using high-frequency stimulation (~100 Hz) was found to restore spine density and long-term memory in early stages of the disease in mice (Roy et al., 2016). While these findings are specific to Alzheimer’s disease, they also confirm an important link between gamma synchrony and cognitive function exists (Fries, 2015). Furthermore, we may find disruptions in high-frequency oscillation patterns tightly correspond with the degree of cognitive impairment and range of pathologies across *EHMT1* risk populations.

The decreases in high-frequency activity coupled with reduced *Grin1* mRNA are suggestive of overall disruptions in local connectivity and may hint at more global imbalances in excitation/inhibition (E/I). Such findings do corroborate previous reports showing abnormalities in neural development and connectivity in *Ehmt1*^+/−^ mouse models (Balemans et al., 2013, 2014; Benevento et al., 2017), and that *Ehmt1-*mediated H3K9me2 levels dynamically regulate synaptic scaling, thus playing a direct role in the fine balance between excitation and inhibition at the level of individual neurons (Benevento et al., 2016). And indeed, many of the *Ehmt1*^D6Cre/+^ mice phenotypes are markedly similar to *Grin1* mouse mutants (Furuse et al., 2010). The *Grin1*^neo−/−^ mice and the *Grin1*^Rgsc174^ heterozygous mice both show increased stereotypy (Mohn et al., 1999) and deficits in sensorimotor gating (Duncan et al., 2004). Mice with even subtle reductions in the NR1 receptor are found to have decreases in MMN (Featherstone et al., 2015), gamma-band disruptions and reduction in E/I balance (Gandal et al., 2012). Nevertheless, it is important to note that the studies presented here represent a change in mRNA and not protein levels and, moreover, only provide a correlative link between the electrophysiological deficits seen in *Ehmt1*^D6Cre/+^ mice and *Grin1* reduction. Interestingly however, a recent study has demonstrated *increased Grin1* expression and NMDA-R hyperactivity in iPSCs derived from Kleefstra syndrome patients (Frega et al., 2019). Despite the discrepancies, together these data suggest that future investigation of *Ehmt1* haploinsufficiency may benefit from further examination of the relationship with the NMDA system.

In summary, *Ehmt1*^D6Cre/+^ forebrain-specific haploinsufficiency produced deficits in sensory-gating and information processing. Behavioural evidence from explicit tests of sensorimotor gating and findings from a learning and memory task, suggest that *Ehmt1*^D6Cre/+^ mice do not attend to or process information in order to inform appropriate behavioural responses. Neural correlates of these abnormalities were further demonstrated using electrophysiological studies, which indicated deficits related to disruptions in local connectivity and NMDA function. Taken together these data suggest that *Ehmt1* haploinsufficiency leads to abnormal circuit formation and behavioural abnormalities that likely underpin deficits seen across the broad spectrum of neurodevelopmental and neuropsychiatric disorders with which *EHMT1* is associated.

## Supplemental Material

Davis_et_al_EPOCH – Supplemental material for Impairments in sensory-motor gating and information processing in a mouse model of Ehmt1 haploinsufficiency Click here for additional data file. Supplemental material, Davis_et_al_EPOCH for Impairments in sensory-motor gating and information processing in a mouse model of Ehmt1 haploinsufficiency by Brittany A Davis, François David, Ciara O’Regan, Manal A Adam, Adrian J Harwood, Vincenzo Crunelli and Anthony R Isles in Brain and Neuroscience Advances This article is distributed under the terms of the
Creative Commons Attribution 4.0 License ( http://www.creativecommons.org/licenses/by/4.0/)
which permits any use, reproduction and distribution of
the work without further permission provided the original
work is attributed as specified on the SAGE and Open
Access pages ( https://us.sagepub.com/en-us/nam/open-access-at-sage).

Davis_et_al_NOR – Supplemental material for Impairments in sensory-motor gating and information processing in a mouse model of Ehmt1 haploinsufficiency Click here for additional data file. Supplemental material, Davis_et_al_NOR for Impairments in sensory-motor gating and information processing in a mouse model of Ehmt1 haploinsufficiency by Brittany A Davis, François David, Ciara O’Regan, Manal A Adam, Adrian J Harwood, Vincenzo Crunelli and Anthony R Isles in Brain and Neuroscience Advances This article is distributed under the terms of the
Creative Commons Attribution 4.0 License ( http://www.creativecommons.org/licenses/by/4.0/)
which permits any use, reproduction and distribution of
the work without further permission provided the original
work is attributed as specified on the SAGE and Open
Access pages ( https://us.sagepub.com/en-us/nam/open-access-at-sage).

Davis_et_al_qPCR – Supplemental material for Impairments in sensory-motor gating and information processing in a mouse model of Ehmt1 haploinsufficiency Click here for additional data file. Supplemental material, Davis_et_al_qPCR for Impairments in sensory-motor gating and information processing in a mouse model of Ehmt1 haploinsufficiency by Brittany A Davis, François David, Ciara O’Regan, Manal A Adam, Adrian J Harwood, Vincenzo Crunelli and Anthony R Isles in Brain and Neuroscience Advances This article is distributed under the terms of the
Creative Commons Attribution 4.0 License ( http://www.creativecommons.org/licenses/by/4.0/)
which permits any use, reproduction and distribution of
the work without further permission provided the original
work is attributed as specified on the SAGE and Open
Access pages ( https://us.sagepub.com/en-us/nam/open-access-at-sage).

Davis_et_al_Rotarod – Supplemental material for Impairments in sensory-motor gating and information processing in a mouse model of Ehmt1 haploinsufficiency Click here for additional data file. Supplemental material, Davis_et_al_Rotarod for Impairments in sensory-motor gating and information processing in a mouse model of Ehmt1 haploinsufficiency by Brittany A Davis, François David, Ciara O’Regan, Manal A Adam, Adrian J Harwood, Vincenzo Crunelli and Anthony R Isles in Brain and Neuroscience Advances This article is distributed under the terms of the
Creative Commons Attribution 4.0 License ( http://www.creativecommons.org/licenses/by/4.0/)
which permits any use, reproduction and distribution of
the work without further permission provided the original
work is attributed as specified on the SAGE and Open
Access pages ( https://us.sagepub.com/en-us/nam/open-access-at-sage).

Davis_et_al_Startle_and_PPI – Supplemental material for Impairments in sensory-motor gating and information processing in a mouse model of Ehmt1 haploinsufficiency Click here for additional data file. Supplemental material, Davis_et_al_Startle_and_PPI for Impairments in sensory-motor gating and information processing in a mouse model of Ehmt1 haploinsufficiency by Brittany A Davis, François David, Ciara O’Regan, Manal A Adam, Adrian J Harwood, Vincenzo Crunelli and Anthony R Isles in Brain and Neuroscience Advances This article is distributed under the terms of the
Creative Commons Attribution 4.0 License ( http://www.creativecommons.org/licenses/by/4.0/)
which permits any use, reproduction and distribution of
the work without further permission provided the original
work is attributed as specified on the SAGE and Open
Access pages ( https://us.sagepub.com/en-us/nam/open-access-at-sage).

Davis_et_al_Suppl_Info_Revision – Supplemental material for Impairments in sensory-motor gating and information processing in a mouse model of Ehmt1 haploinsufficiency Click here for additional data file. Supplemental material, Davis_et_al_Suppl_Info_Revision for Impairments in sensory-motor gating and information processing in a mouse model of Ehmt1 haploinsufficiency by Brittany A Davis, François David, Ciara O’Regan, Manal A Adam, Adrian J Harwood, Vincenzo Crunelli and Anthony R Isles in Brain and Neuroscience Advances
